# Resveratrol Attenuates CSF Markers of Neurodegeneration and Neuroinflammation in Individuals with Alzheimer’s Disease

**DOI:** 10.3390/ijms26115044

**Published:** 2025-05-23

**Authors:** Xiaoguang Liu, Sean Baxley, Michaeline Hebron, Raymond Scott Turner, Charbel Moussa

**Affiliations:** 1 Laboratory for Dementia and Parkinsonism, Translational Neurotherapeutics Program, Department of Neurology, Georgetown University Medical Center, Washington, DC 20057, USA; xl371@georgetown.edu (X.L.); sdb82@georgetown.edu (S.B.); mlh88@georgetown.edu (M.H.); 2 Interdisciplinary Program in Neuroscience, Georgetown University Medical Center, Washington, DC 20057, USA; 3 Memory Disorders Program, Department of Neurology, Georgetown University, Washington, DC 20057, USA; rst36@georgetown.edu

**Keywords:** Alzheimer’s disease, resveratrol, neurodegeneration, neuroinflammation

## Abstract

Alzheimer’s disease (AD) is a progressive neurodegenerative disorder that is characterized by amyloid-beta (Aβ) accumulation and neuroinflammation. A previous multicenter, phase 2, double-blind, placebo-controlled trial randomized 179 participants into placebo or resveratrol over 52 weeks. Sub-analysis of CSF biomarkers of neuronal damage, inflammation, and microglial activity was performed in a subset of patients treated with a placebo (n = 21) versus resveratrol (n = 30). Markers of neuronal damage, including neuron-specific enolase and hyperphosphorylated neurofilaments, were reduced. Microglial activation was measured via a triggering receptor expressed on myeloid cells (TREM)-2 at baseline and after resveratrol treatment. Resveratrol significantly reduced CSF TREM2 levels and decreased inflammation and tissue damage, including matrix metalloprotease (MMP)-9. Cathepsin D, a lysosomal marker of autophagy, was reduced in the resveratrol group compared with placebo, while angiogenin, a marker of vascular angiogenesis, was increased. These data suggest that resveratrol may exert anti-inflammatory and neuroprotective effects in AD by reducing CSF TREM2 and other markers of neuronal damage. Further research is needed to assess the significance of these biomarker changes on clinical outcomes in patients with neurodegenerative diseases.

## 1. Introduction

Alzheimer’s disease (AD) is the most common form of dementia, affecting millions of people worldwide. One of the pathological hallmarks of AD is accumulation of amyloid-beta (Aβ) plaques and neurofibrillary tangles composed of hyperphosphorylated tau in the brain. These events are associated with chronic neuroinflammation [1], which may promote disease progression and severity. Microglia serve as the resident immune cells of the brain, and their activation is crucial for clearing cellular debris, including Aβ. However, dysregulated microglial activity has been implicated in the progression of AD, as overactivated microglia can contribute to chronic inflammation and exacerbate neuronal damage [2]. There is a growing interest in the role of microglial cells, which play a key role in modulating the inflammatory response in AD [3]. Given the complex interplay between microglial activity, Aβ aggregation, and neuroinflammation, it is imperative to explore therapeutic modulators of these pathways.

Resveratrol is a sirtuin-1 (SIRT1) activator that stimulates mitochondrial biogenesis [4,5,6,7,8,9,10] and regulates autophagy [11,12]. Resveratrol facilitates mitophagy [13], which is necessary for mitochondrial dynamics, which is deficient in neurodegenerative diseases [14,15]. Resveratrol also inhibits the activation of the NLRP3 inflammasome (also known as nucleotide-binding domain, leucine-rich repeat, and pyrin domain-containing protein 3) and lowers inflammation [16,17]. The various roles of resveratrol to inhibit the NLRP3 inflammasome, promote autophagy, and maintain mitochondrial integrity [18] suggest that resveratrol is a therapeutic candidate that has strong neuroprotective effects in AD and other neurodegenerative diseases [17,19].

A previous phase 2, multicenter, placebo-controlled, clinical trial investigated the effects of resveratrol on biomarkers for cognitive and functional outcomes in patients diagnosed with mild-to-moderate AD [20]. Post hoc analysis of cerebrospinal fluid (CSF) samples from these patients showed that treatment with resveratrol stabilized CSF Aβ levels and reduced markers of neuroinflammation, including matrix metalloprotease (MMP)-9, and regulated microglial activity [21]. This report further investigates the role of resveratrol on autophagy via the lysosomal marker cathepsin D and microglial activation via a triggering receptor expressed on myeloid cells (TREM-2), as well as other CSF biomarkers of neuronal and vascular damage in AD patients.

## 2. Results

### 2.1. Resveratrol Reduces CSF Markers of Cell Death

**Figure 1:**
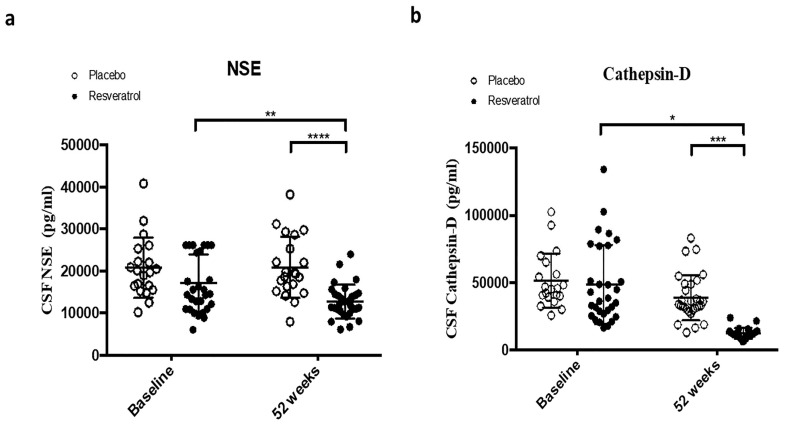
ELISA measurements show the concentrations of (**a**) Neuron specific enolase (NSE) and (**b**) Cathepsin-D that are significantly reduced in the CSF of patients treated with resveratrol (n = 30) compared to placebo (n = 21) ta baseline and 52 weeks. Mean ± SD, *p*-values and statistical methods are listed in Table 1.

**Figure 2:**
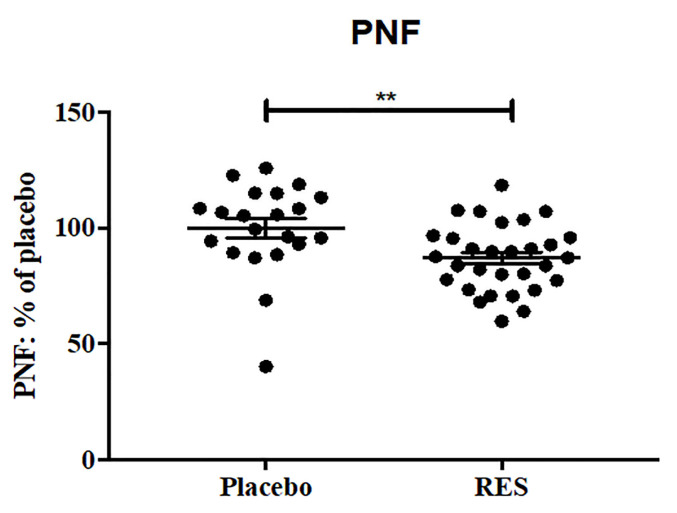
ELISA measurement of phosphorylated neurofilaments (PNF) concentrations expressed as percentages normalized to placebo group shows a significant reduction in PNF in the CSF of patients treated with placebo (n = 21) compared to resveratrol (RES, n = 30) for 52 weeks. Mean ± SD, *p* values and statistical methods are listed in Table 1.

We first studied the effects of resveratrol treatment on molecular markers of cell death and autophagy in the CSF of AD patients. At baseline, there were no significant differences in CSF levels of neuron-specific enolase (NSE) or cathepsin D between the placebo and resveratrol groups (Figure 1a, Table 1). After 52 weeks, the resveratrol group showed a significant reduction in CSF NSE levels compared with baseline (Figure 1a). Notably, NSE levels in the resveratrol group were reduced by 40% compared with the placebo group at 52 weeks (Figure 1a). NSE is a well-established biomarker of cerebral tissue damage and is commonly used to assess stroke severity [22,23,24,25,26]. In addition, CSF levels of cathepsin D were significantly lower in the resveratrol group at 52 weeks compared with both baseline and the placebo group (Figure 1b, Table 1). Cathepsin D, a lysosomal proteinase, is highly expressed in senile plaques and typically elevated in the CSF of AD patients [27,28,29]. The effects of resveratrol on phosphorylated neurofilaments (PNF) were also measured (Table 1, Figure 2). The percentages of PNF at 52weeks are expressed relative to baseline because the PNF standards were not reliable. At week 52, PNF was significantly reduced in the resveratrol group compared with placebo.

### 2.2. CSF Biomarker of Angiogenesis and TREM2

**Figure 3:**
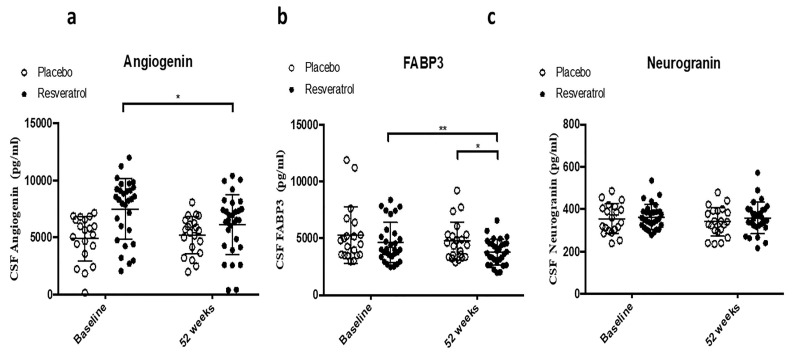
ELISA concentrations of (**a**) Angiogenin, (**b**) Fatty Acid Binding Protein 3 (FABP3), and (**c**) Neurogranin in the CSF of patients treated with placebo (N = 21) or resveratrol (N = 30) for 52 weeks. Mean ± SD, *p* values and statistical methods are listed in Table 1.

We then investigated the effects of resveratrol treatment on molecular markers of inflammation and angiogenesis in the CSF of AD patients. The levels of angiogenin, a biomarker of angiogenesis, significantly decreased within the resveratrol group by 52 weeks (Figure 3a, Table 1). Baseline levels of fatty acid binding protein 3 (FABP3) (Figure 3b, Table 1) and neurogranin (Figure 3c, Table 1) in the CSF were similar between the placebo and resveratrol groups. Over 52 weeks, FABP3 levels were significantly reduced in the resveratrol group compared with both baseline and the placebo group at 52 weeks (Figure 3b). No significant changes were observed in CSF neurogranin levels across groups (Figure 3c).

### 2.3. CSF Biomarkers of Matrix Metalloproteases as Dual Markers of Inflammation and BBB Integrity

**Figure 4:**
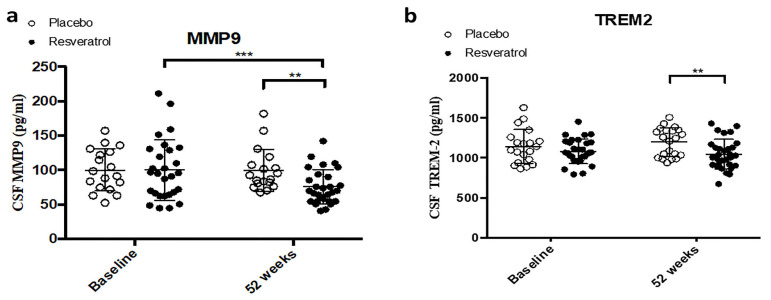
ELISA measurement shows concentrations of (**a**) matrix metalloprotease (MMP)-9 is significantly reduced in the CSF of patients treated with resveratrol (N = 30) compared to placebo (N = 21) for 52 weeks. (**b**) Triggering Receptor Expressed on Myeloid Cells (TREM)-2 is significantly reduced in the CSF of patients treated with resveratrol (N = 30) compared to placebo (N = 21) for 52 weeks. Mean ± SD, *p* values and statistical methods are listed in Table 1.

We assessed the effects of resveratrol treatment on the CSF levels of MMPs and tissue inhibitor of metalloproteinases (TIMPs) as markers of possible vascular damage and neuroinflammation. At baseline, CSF levels of MMP-9 and TREM2 were not significantly different between the placebo and resveratrol groups (Table 1, Figure 4). After 52 weeks, MMP-9 levels were significantly reduced in the resveratrol group compared with both baseline and the placebo group (Figure 4a, Table 1). Similarly, TREM2 levels were significantly lower in the resveratrol group compared with the placebo group at 52 weeks (Figure 4b, Table 1).

**Figure 5:**
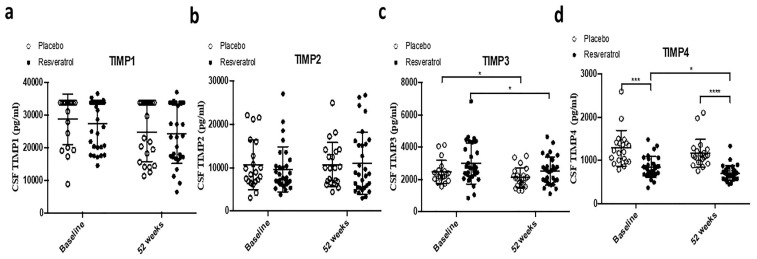
ELISA measurements of concentrations of (**a**) Tissue Inhibitor of Metalloproteinase (TIMP)-1, (**b**) TIMP2, (**c**) TIMP3, and (**d**) TIMP4 in the CSF of patients treated with placebo (N = 21) or resveratrol (N = 30) for 52 weeks. Mean ± SD, *p* values and statistical methods are listed in Table 1.

TIMPs 1, 2, 3, and 4 were also measured. No significant differences in TIMP-1, TIMP-2, or TIMP-3 levels were observed between groups at baseline (Figure 5a–c, Table 1). By 52 weeks, TIMP-3 levels were significantly reduced in both the resveratrol and placebo groups compared with baseline (Figure 5c). TIMP-4 levels, which were significantly different at baseline between the two groups, showed a significant reduction within the resveratrol group by 52 weeks (Figure 5d).

## 3. Discussion

Resveratrol decreases aging-dependent cognitive decline and pathology in animal models of AD [30]. Preclinical studies have shown that resveratrol can reduce Aβ aggregation, protect neurons from oxidative stress, and attenuate neuroinflammation and its ability to cross the blood–brain barrier (BBB), which further supports its potential as a treatment for neurodegeneration [20,31,32]. Resveratrol can also be relevant in chronic diseases and longevity in humans [33,34]. A randomized, placebo-controlled, double-blind, multicenter, 52-week trial of resveratrol in individuals with mild-to-moderate AD reported its safety and tolerability [20]. Doses increased in 500 mg increments every 13 weeks, but no differences between the resveratrol- and placebo-treated groups were found in laboratory tests, vital signs, physical examinations, or neurologic examinations. Further ad hoc analysis in an enriched population with a pre-specified biomarker, including low CSF Aβ42 levels, showed that CSF Aβ and microglial activity were significantly reduced after 52 weeks of resveratrol treatment [21]. The sub-analysis also suggested that biomarker changes are associated with functional improvement in activities of daily living in AD patients [21]. Cathepsin D is a lysosomal protein that contributes to autophagy in many neurodegenerative diseases, and its levels are elevated in the CSF of patients with AD, suggesting it could be a potential biomarker of autophagy [35,36]. Our data show a significant decrease in cathepsin D after 1 year of treatment with resveratrol. In addition to resveratrol effects on lysosomal clearance and regulation of microglial function, a decrease in markers of axonal damage, NSE [22,37], and phosphorylated neurofilaments further suggests a neuroprotective role of resveratrol. Neurogranin is a protein primarily found in dendritic spines and contributes to synaptic plasticity, and its levels are elevated in the CSF of AD patients compared with controls [38], but no change in CSF levels of neurogranin was observed with resveratrol treatment. The role of cathepsin D is complex, and its elevated levels in AD may indicate a compensatory effect to regulate autophagy, but the reduction in biomarkers of axonal damage together with cathepsin D suggest regulation of autophagy and attenuation of neuronal damage.

Overactivated microglia contribute to chronic inflammation and exacerbate neuronal damage [2]. Our data indicate a significant decrease in CSF levels of TREM2 after resveratrol treatment. TREM2 is a transmembrane receptor that binds to various ligands and contributes to microglia clearance of cellular debris, including Aβ plaques, thereby regulating microglial function and clearance [39]. Soluble TREM2 (sTREM2) is released from cell membranes and binds Aβ to prevent its aggregation, suggesting a protective role against AD [40]. Carriers of TREM2 mutations exhibit increased amyloid deposition and accelerated cognitive decline [40], perhaps due to a dysregulated microglial response to Aβ [41]. In fact, TREM2 is upregulated in response to Aβ accumulation, and it facilitates microglial clearance of amyloid plaques. However, sustained TREM2 activation can exacerbate inflammatory responses and tissue damage [42]. TREM2 has gained attention due to its involvement in various neurodegenerative diseases, including AD, multiple sclerosis, amyotrophic lateral sclerosis (ALS), and Parkinson’s disease (PD). TREM2 promotes microglial survival, migration, and phagocytosis of apoptotic cells, making it a critical regulator of neuroinflammatory processes in the brain [43]. Given the beneficial interplay between TREM2, microglial activity, and neuroinflammation, it is beneficial to explore the potential of resveratrol as a common therapeutic modulator of these pathways. TREM2-targeted therapies could provide a novel approach to balancing microglial activation, reducing harmful inflammation, and promoting toxic protein clearance.

Tissue inhibitors of metalloproteinases (TIMPs) are a family of proteins that inhibit the activity of MMPs to prevent the degradation of the extracellular matrix (ECM) and prevent tissue damage and inflammation [44]. Resveratrol reduces CSF levels of both TIMP-3 and TIMP-4, as well as MMP-9, consistent with our previous data [21], suggesting potential reduction of inflammation in AD patients. The role of TIMPs and MMPs, such as MMP-9, is critical in both inflammation and vascular changes, including angiogenesis in neurodegeneration [45]. Angiogenin is a ribonuclease, which plays a crucial role in angiogenesis and formation of new blood vessels in stress responses, and has been linked to various diseases, including neurodegeneration [38]. Our data show a decrease in TIMPs and MMP-9, suggesting reduced vascular and ECM damage, while the increase in the level of angiogenin may contribute to potential vascular remodeling after resveratrol treatment. However, the manifestation of changes in TIMPs and MMPs is varied in different diseases and different tissues. For example, elevated levels of MMP-2, MMP-3, MMP-10, TIMP-1, and TIMP-2 were detected in the CSF of patients with delirium who had pre-existing dementia [46]. Conversely, MMP-2, MMP-12, and TIMP-1 were higher in acute trauma (e.g., hip fracture) compared with healthy controls or patients without dementia. In a totally unrelated condition such as pyramidal syndrome or progressive weakness and stiffness of the lower limbs due to tropical spastic paraparesis and viral infection (inflammation), TIMP-2, TIMP-3, and TIMP-4 levels were increased compared with healthy controls, while TIMP-1 was only increased in the presence of inflammation, and MMP-3 and MMP-9 levels were significantly increased in the presence or absence of inflammation [47]. MMPs as a family of proteinase can play critical roles in acute and chronic neuroinflammation, as well as blood vessel integrity and BBB permeability [48,49]. The variable levels of MMPs and/or TIMPs may be due to their proteolytic substrates that may also depend on the tissue as well as inflammatory conditions, blood vessel status, and specific processing stage of the proteolytic activity of these molecules, thus providing differential read-out and relationship in different conditions.

Resveratrol could also be a candidate for the treatment of traumatic brain injury (TBI), which is a major cause of death and disability [50], and individuals who experience TBI are at increased risk for several neurological disorders, such as PD, ALS, MS Lewy body dementia (LBD), and AD [51]. The potential efficacy of resveratrol to treat TBI has been demonstrated in preclinical TBI models [52]. Resveratrol attenuates the level of molecular markers of damage, including malondialdehyde (MDA), glutathione (GSH), nitric oxide (NO), and xanthine oxidase (XO) [53], therefore providing a neuroprotective strategy against brain damage. Resveratrol was also shown to halt cognitive deficits that are mediated via p38 activation and subsequent activation of the nrf2 (nuclear factor 1 erythroid nuclear factor 2) pathway in TBI rats [54]. Resveratrol was also shown to lower neuroinflammation and enhance long-term hippocampal functioning in models of TBI [55]. Aside from these specific examples in TBI models, resveratrol has neuroprotective effects in many disease conditions [20,21,32,56], and it is imperative to explore the role of resveratrol as a preventative strategy in neurodegeneration.

Resveratrol’s neuroprotective effects are mediated through multiple mechanisms, including the activation of SIRT1, a protein deacetylase involved in cellular stress responses. In addition, resveratrol exerts anti-inflammatory effects by inhibiting the nuclear factor kappa-light-chain-enhancer of activated B cells (NF-κB) signaling pathway, a central regulator of inflammation [57,58]. By targeting these molecular pathways, resveratrol may help reduce chronic inflammation that contributes to the progression of AD. ABP3 (also known as heart-type fatty acid binding protein) transports fatty acids to the mitochondria, facilitating their use for energy production and regulation of energy balance by controlling fatty acid metabolism [59] and mitochondrial autophagy [60]. Biologically, resveratrol stimulates mitochondrial biogenesis [4,5,6,7,8] and regulates mitochondrial dynamics via autophagy [11,12]. Resveratrol plays a role in parkin-related mitophagy [13], which is necessary for mitochondrial dynamics, but it is deficient in neurodegenerative diseases, including PD and AD [14,15].

PD is the second most common neurodegenerative disorder causing motor and non-motor symptoms, loss of dopamine (DA), and aggregation of misfolded alpha-synuclein [61,62,63]. There is evidence of the presence of oxidative stress and mitochondrial damage in the brains of patients with PD [64,65,66]. Specific gene mutations that cause PD can cause oxidative stress and mitochondrial dysfunction in the familial and the sporadic forms of PD [64,65,66]. PD-linked familial gene mutations, like PTEN-induced putative kinase 1 (PINK1), DJ-1, alpha-synuclein, leucine-rich repeat kinase 2 (Lrrk2), and parkin, express proteins that interact with mitochondrial proteins, oxidative stress, and free radicals [67,68,69]. The SIRT1 activator resveratrol promotes mitochondrial biogenesis [4,5,6,7,8] and regulates mitochondrial dynamics via mitophagy [11,12]. The role of mitochondrial energy metabolism, including production of adenosine triphosphate (ATP) and nicotinamide adenine dinucleotide (NAD+), plays a critical role in the etiology and pathogenesis of PD [64,65,66], therefore optimizing a therapeutic option that enhances energy metabolism and may counteract alpha-synuclein aggregation and attenuate inflammation [67,68,69]. ATP and NAD^+^ are deficient in PD and several other neurodegenerative diseases [70], and mitochondria produce ATP to meet energy demands, regulate homeostasis, and lower reactive oxygen species that cause oxidative stress and loss of DA in PD [71,72,73]. Dopaminergic neurons seem to be vulnerable to the toxic events that occur during the neurodegenerative process of PD, and mitochondrial energy metabolism and integrity are central to these pathways [74]. Mitochondrial ATP production is deficient in muscles of PD patients [75]. Resveratrol may improve energy metabolism and have the potential to slow PD progression [76,77]. Mitochondria are essential for neuronal function, and enhancement of glycolysis increases ATP levels and may lead to novel therapeutic options for PD [78].

Friedreich’s ataxia (FRDA) is another autosomal-recessive inherited neurodegenerative disease, and the most common ataxia caused by mutation in the *FXN gene*, resulting in deficiency of the mitochondrial protein frataxin. Resveratrol increases frataxin expression in vitro and in vivo in models of FRDA. A 1.5- to 2-fold increase in frataxin protein expression was observed in lymphoblasts and fibroblasts derived from individuals with FRDA, and a 200 mg/kg subcutaneous RSV resulted in a 1.5-fold increase in human frataxin protein in the brain of humanized FRDA (YG8R) mice [79]. An open-label study in 24 individuals with FRDA showed clinical improvements via composite Friedreich’s ataxia endpoints and benefits to speech and hearing following treatment with resveratrol, as well as a reduction in plasma F2-isoprostanes, a marker of oxidative stress, compared with baseline [80]. Mucopolysaccharidosis type I (MPS I) is an autosomal-recessive disease that presents with chronic, progressive, multisystem, lysosomal storage caused by deficiency or lack of activity of the α-L-iduronidase (IDUA) enzyme [81,82,83,84], and resveratrol was shown to upregulate IDUA activity [85], providing a potential treatment option for MPSI.

There is a critical need to develop new disease-modifying therapies for neurodegenerative diseases, including AD and PD. One promising candidate for such modulation is resveratrol, a naturally occurring polyphenol. Resveratrol is an antioxidant, anti-inflammatory, and neuroprotective agent, and it has been explored as a potential therapeutic agent for neurodegenerative diseases due to its multifaceted biological roles [34,86].

## 4. Materials and Methods

### 4.1. Patient Demographics

With the Alzheimer’s Disease Cooperative Study (ADCS), a randomized, placebo-controlled, double-blind, multi-site, phase 2 trial of resveratrol in individuals with mild-to-moderate dementia due to AD was previously completed [20]. The study drug was pure, synthetic, resveratrol powder (encapsulated) versus a matching placebo. Concomitant use of FDA-approved medications for AD (e.g., cholinesterase inhibitors) was allowed. Participants (total N = 179) were randomized to placebo or resveratrol with 500 mg orally once daily (with a dose increase of 500 mg increments every 13 weeks, ending with 1000 mg twice daily). The total treatment duration was 52 weeks. A total of N = 56 participants completed the trial in the resveratrol arm, and N = 48 completed week 52 in the placebo. The primary outcomes were safety and tolerability and resveratrol effects on AD biomarkers (Aβ40 and Aβ42, CSF Aβ40, Aβ42, tau, and phospho-tau181) and volumetric MRI (primary outcomes). Clinical outcomes (secondary) were also examined. Detailed pharmacokinetics were obtained in a subset (n = 15) at baseline and at weeks 13, 26, 39, and 52. Resveratrol entered the brain, as its major metabolites were detected in plasma and CSF. Weight loss was observed in the resveratrol group. Compared with a decline found in the placebo group, plasma Aβ40 and CSF Aβ40 levels were stabilized by resveratrol. In the subset of individuals with biomarker-confirmed AD (baseline Aβ42 < 600 ng/mL), resveratrol treatment also stabilized CSF Aβ42. Brain volume loss was increased by resveratrol treatment (3 versus 1%), suggesting a potent anti-inflammatory effect [21].

### 4.2. Multiplex Xmap ELISA

Xmap technology uses magnetic microspheres that are internally coded with two fluorescent dyes. Through precise combinations of these two dyes, multiple proteins are simultaneously measured within a sample. All samples, including placebo and resveratrol at baseline and 52 weeks, were analyzed in parallel using the same reagents. A total of 25 μL of human CSF or plasma was incubated overnight at 4 °C with 25 μL of a mixed bead solution containing MMP-1, -2, -7, -9, and -10 (Millipore Cat# HMMP1MAG-55K, Rockville, MD, USA), MMP-3, -12, and -13. (Millipore Cat# HMMP2MAG-55K), tissue inhibitor of metalloproteases TIMP-1 and -2 (Millipore Cat# HTIMP1MAG-54K), TIMP-1, -2, -3, and -4. (Millipore Cat# HTIMP2MAG-54K), Cathepsin D (Millipore Cat# HNDG3MAG-36K), neuron-specific enolase -NSE (Millipore Cat# HNDIMAG-39K), and a panel including angiogenin, FABP3 (fatty acid binding protein 3), TREM 2, and neurogranin (Millipore Cat# HNS2MAG-95K). After washing, samples were incubated with 25 μL of detection antibody solution for 1.5 h at room temperature. Streptavidin-phycoerythrin (25 μL) was added to each well containing the 25 μL of detection antibody solution. Samples were then washed and suspended in 100 μL of sheath fluid. Samples were then run on MAGPIX with Xponent software 4.3 (Millipore 40-012, Luminex200^TM^ System). The median fluorescent intensity (MFI) data was analyzed using a 5-parameter logistic or spline curve-fitting method for calculating analyte concentrations in samples.

### 4.3. Standard Protocol Approvals, Registrations, and Patient Consent

This study was conducted in accordance with Good Clinical Practice guidelines. Informed consent was obtained from participants and study partners. The study was conducted under local institutional review board supervision, under Food and Drug Administration IND 104205, and registered at ClinicalTrials.gov (NCT01504854).

### 4.4. Statistical Analysis

The outcomes measured here are all exploratory, post hoc analyses. Data are summarized as raw values, range as appropriate, and mean ± SD for N = 21 in the placebo group and N = 30 in the resveratrol group. All graphs and statistical analyses were performed in Graph Pad Prism Software version 7.0 (Graph Pad Prism Software, Inc., La Jolla, CA. USA). For baseline comparison between the two treatment arms, unpaired *t*-tests assuming both equal and unequal variances and Wilcoxon rank sum tests, were performed to compare biomarkers and clinical variables. Paired *t*-tests were performed within groups at baseline versus 52 weeks of treatment, and unpaired *t*-tests were performed for comparison of placebo and resveratrol treatments.

## Figures and Tables

**Table 1:** Summary of statistical tests of null changes between baseline and 52 weeks and tests of null differences at baseline and at 52 weeks using all detected molecules in CSF of patients treated with placebo (N = 21) or resveratrol (N = 30). Statistical significance: * *p* < 0.05, ** *p* < 0.01, *** *p* < 0.001, and **** *p* < 0.0001.

Analytes	Baseline 52 Weeks				Baseline		52 Weeks	
	Paired *t*-Test		Wilcoxon Signed-Rank Test		Baseline: Active vs. Placebo		52 Weeks: Active vs. Placebo	
	Placebo	Resveratrol	Placebo	Active	Unpaired *t*-Test (Unequal)	Unpaired *t*-Test (Equal)	Unpaired *t*-Test (Unequal)	Unpaired *t*-Test (Equal)
NSE	0.8594	0.0051 **	0.3371	0.0047 **	0.0387 *	0.0773	<0.0001 ****	<0.0001 ****
Cathepsin D	0.2053	0.0971	0.1153	0.0701	0.6863	0.7071	0.0002 ***	0.0001 ***
TREM2	0.3622	0.2754	0.1153	0.1746	0.2755	0.243	0.0023 **	0.0048 **
Angiogenin	0.5284	0.0437 *	0.4492	0.0249 *	0.0002 ***	0.0005 ***	0.0636	0.1581
FABP3	0.2216	0.0066 **	0.252	0.0027 **	0.3111	0.2762	0.164 *	0.0205 *
Neurogranin	0.3955	0.777	0.2152	0.2274	0.6684	0.656	0.2013	0.41
p-NF	NA	NA	NA	NA	NA	NA	0.0131 *	0.0081 **
MMP-2	0.1692	0.6759	0.229	0.8067	NA	NA	NA	NA
MMP-9	0.3621	0.0003 ***	0.3778	0.0004 ***	>0.9999	>0.9999	0.0092 **	0.0058 **
MMP-10	0.064	0.2527	0.0843	0.1502	NA	NA	NA	NA
TIMP-1	0.1177	0.1368	0.0946	0.0698	0.5233	0.5187	0.8294	0.8293
TIMP-2	0.9452	0.3568	0.8983	0.6702	0.4965	0.4867	0.8517	0.8600
TIMP-3	0.0275 *	0.0371 *	0.0153 *	0.0323 *	0.3613	0.3692	0.0504	0.0688
TIMP-4	0.0968	0.0126 *	0.0583	0.0086 **	0.0002 ***	<0.0001 ****	<0.0001 ****	<0.0001 ****

## Data Availability

The final data generated and analyzed, materials, and all interpretations are available to the scientific and non-scientific community upon request. No datasets are deposited in a repository, and there is no web link to any datasets.
